# PROTOCOL: Effects of interventions for infant and young child feeding (IYCF) promotion on optimal IYCF practices, nutrition, growth and health in low‐ and middle‐income countries: a systematic review

**DOI:** 10.1002/CL2.189

**Published:** 2018-12-20

**Authors:** Zohra S Lassi, Omar Irfan, Rabia Hadi, Jai K Das, Zulfiqar A Bhutta

## Background

### The problem, condition or issue

According to the World Health Organization (WHO), under‐nutrition is associated with 2.7 million child deaths worldwide (Rollins 2016). This accounts for 45% of infant deaths globally, among which low‐ and middle‐income countries (LMICs) bear the greatest burden (UNICEF 2017). About 99% of this burden is shared by LMIC from Sub‐Saharan Africa and South Asia (WHO 2012). Infant malnutrition leads to an increase in the frequency and severity of common infections which raises the risk of death during a vulnerable stage in life (UNICEF 2016). Under‐nutrition compromises immunity with increased susceptibility to infections and hastens the severity of disease. These infections can increase energy requirements with reduced appetite and absorption of nutrients leading to poor health outcomes (Bhutta 2013). Therefore, LMICs may face a greater problem in treating these medical concerns due to a lack of medical resources which further increases the risk of child deaths in medically underserved areas. Moreover, in LMICs, an estimated 250 million children under‐five are at risk of sub‐optimal development and stunting or low height‐for‐age (Rollins 2016). Nutrition is not only essential for the infant's health and physical development but also plays a major role in cognitive development which has a lasting effect on school and work performance later in life (UNICEF 2016). Stunting itself is associated with poor physical and cognitive development with low performance in schools. It is also known to affect the economic productivity of adults and Gross Domestic Product (GDP) of the country. Stunted children are also at a higher risk of mortality (Olofin 2013); wasting, on the other hand, can predispose children to life‐threatening infections as well because of secondary immunodeficiency. Moreover, wasting likely contributes to stunting and other forms of developmental impairments (Menon 2012).

Lack of essential nutrition within an infant's diet can lead to life‐threatening conditions known as moderate‐ and severe‐ acute malnutrition (MAM/SAM). Acute malnutrition is classified as mild, moderate or severe based on anthropometric measurements affecting an estimated 51.5 million children under‐five years of age and being associated with 12.6% of all under five infant mortality (James 2016). SAM is defined by a very low weight for height score (below ‐3 Z scores of the median WHO growth standards), with visible severe wasting and/or presence of nutritional edema (WHO 2009). While MAM is defined by a lesser degree of wasting from similar causes as SAM, it bears a greater prevalence of 64% of all those characterized with acute malnutrition (James 2016). In 2011, 16% of infants globally had a weight for age below ‐2 Standard Deviation (SD) and 8% had a weight for height below ‐2SD, characterized as underweight and wasted respectively (UNICEF 2012). With these statistics, acute malnutrition continues to be a major global health problem.

Maternal breastfeeding and complementary foods have been the most common and accessible modes of providing children with good nutrition during the early years of life. Breast milk has been noted to provide the most optimal nutrition for infants. This is because the components of breast milk including antibodies, hormones and growth factors combined with the nutrition present an optimal diet and protection to the growing child (Michels 2017). Moreover, breast milk is considered a form of personalized medicine for the newborn as it protects growing infants from common infections and diseases and ensures a quick recovery if affected by these health complications (Kramer 2012). It contains a number of components, such as oligosaccharides that inhibit the binding of pathogens and toxins to cell receptors of the host. Oligosaccharides also stimulate the growth of bifidobacteria and lactobacilli that promotes optimal health and immunity of an infant's gut (Zivkovic 2011). In addition to providing immediate energy, breast milk can lead to better long‐term neurodevelopment, and behavioral and cognitive improvement later in life (Michels 2017). Likewise, nursing women benefit from protection against ovarian and breast cancer and prevent diabetes in addition to the physiological benefits that come with breastfeeding (Chowdhary 2015; Jordon 2012). Therefore, it is essential for mothers to implement breastfeeding practices for infant health and development.

After an initial few months of exclusive breastfeeding, infants' dietary requirements increase and are not fulfilled by breastfeeding alone; it is then that infants should be weaned to solid food. An infant's nutritional needs are exponentially growing at this stage and it is essential to provide the required nutrients for optimal growth and development. Infancy is a critical window for growth and after two years of age, reversing stunting and other growth deficiencies that have already occurred becomes extremely difficult (Dewey 2002). Thus, it is essential to provide infants with the required nutritional foods through proper complementary feeding practices. The current picture of complementary feeding adequacy at the regional and global level has been reported (White 2017) using data from the UNICEF global database. Globally, the rates of continued breastfeeding drop from 74.0% at 1 year of age to 46.3% at two years of age. About one‐third of infants who are 4–5 months old are already kept on solid foods, but nearly 20% of 10–11 months old had not consumed solid foods. White also reported that only 28.2% of children aged 6–23 months old, who are receiving at least a minimally diverse diet. Complementary foods can include lipid‐based, ready‐to‐use therapeutic foods, grain and starchy foods, meat, fish, vegetables, eggs, *Khichuri* ‐ which is a traditional mixed dish of rice, lentils, and vegetables. Commonly supplemented foods include Plumpy'doz, Rice and lentil food supplement and fortified wheat‐soy blend to name a few (Campbell 2016). In addition to the health benefits, it is important to acknowledge the economic and environmental advantages that breastfeeding endorses over infant milk substitutes. This is especially important in LMICs because the infant's dietary needs can be addressed with fewer living expenses for families of lower socioeconomic status. Furthermore, breastfeeding and diversification of complementary foods can have a number of environmental benefits which further increases the importance of promoting these practices; this diversity cuts down on containers, packing, fuel to prepare and to transport, and it reduces the carbon cycle by saving global energy and resources (Gartner 2005). Moreover, WHO 2001 has shown that mothers or guardians are willing to prepare culturally acceptable complementary food, and with better knowledge and feeding practices there can be improved dietary intake leading to growth. The messages proposed for education can be improved and modified by integrating suggestions from the mothers. Factors that may influence feeding practices including maternal time contact, her knowledge and attitude, culture, social norms, skills and social influences (Moore 2006).

Despite the vast amount of extant research on infant feeding practices, poor breastfeeding and complementary feeding practices are still the major causes of global child malnutrition (UNICEF 2011). Even now in LMICs only 40% of infants 0‐5 months (Rollins 2016) and 37% of children younger than six months of age (Victora 2016) are exclusively breast‐fed. Furthermore, only two‐thirds of these children are given appropriate solid foods in a timely manner (Rollins 2016). Consequently, it becomes essential for caregivers to receive adequate guidance and education prior to reaching this stage.

### The intervention

It has been reported that a total of 20 countries worldwide have recorded an increase in breastfeeding rates to more than 20% largely through large‐scale implementation of multilevel programs promoting IYCF practices (Child 2011). These interventions aim to promote the health, well being and long‐term development of infants by promoting proper nutrition during the first two years post birth. Interventions can range from a variety of interventions used to implement optimal IYCF practices including:

► Providing direct support and education to mothers and families about proper IYCF practices from a variety of healthcare workers (counsellors, nurses, lactation consultants, midwives, physicians, health care workers) across a range of settings (hospital, home, clinic, community, workplace) and throughout different time periods (prenatal, postpartum).
► Promoting supplementary feeding to prevent moderate and severe acute malnutrition such as endorsing current interventions of preventing SAM/MAM which include tackling the underlying cause of malnutrition through supplementary feeding (James 2016).
► Campaigning programs promoting IYCF through mobile health practices which can include outreach services and peer‐ to‐ peer support groups as reported previously (Prieto 2016).
► Implementing proper training programs to educate healthcare professionals on optimal methods to educate on proper feeding and growth monitoring.

Counseling and education on the importance of breastfeeding can be given in a variety of different ways. Counseling can be given in the antenatal and postnatal periods at home, clinics or in outreach setups. This can be done at the individual level or in a group setting. On the other hand, support that involves encouraging mothers and ensuring the benefits of interventions is usually aimed at an individual level. Complementary feeding involves the initiation of food and liquid to meet the nutritional requirement of infants which are no longer met from breast milk alone. Supplementary feeding involves the provision of energy‐dense food (meals or snacks) or beverages to prevent under‐nutrition. This may be given at home in a community setting, in school, and daycare.

### How the intervention might work

The interventions work to educate mothers and caregivers on infant health and address the barriers that arise in LMICs when engaging in breastfeeding and complementary feeding practices. These interventions aim to promote IYCF guidelines set by the WHO:

► Initiation of maternal breastfeeding within an hour post birth and continuation of exclusive breastfeeding practices until 6 months of age (UNICEF 2011) by educating, counselling and through peer‐support. Appropriate introduction of complementary feeding practices in a timely manner from 6 – 24 months of age. Feeding should be adequate to include appropriate frequency, amounts, consistency and with a variety of foods to address all required infant nutritional needs. This includes proper meal preparation with an emphasis on hygiene and food must be appropriately given to satisfy the consistency and texture, an infant is able to consume depending on age.
► Continuing frequent on‐demand breastfeeding along with supplemental feeding practices from 6‐24 months of age.

The interventions promoting IYCF practices intend to directly increase the number of women and infant caregivers following proper breastfeeding and complementary feeding practices. They also aim to tackle an insufficient diet that may lead to SAM/MAM. This can be implemented through community‐based nutrition education and mobilization programs aiming to increase mother or childcare provider's knowledge in infant feeding practices or with provisional programs to assist families in LMICs with the proper initiation of breastfeeding and complementary feeding practices (Bhutta 2010). Infant feeding practices can also be promoted through counselling programs or support networks which will help mothers during the lactation or feeding period. Financial incentives are another mechanism with which coverage of health and nutrition interventions can be increased (Bassani 2013). We will assess equity by assessing outcomes based on socio‐economic status and wealth where defined and reported in studies. The fidelity to the intervention, duration of intervention, dose delivered and dose received will help us to understand the intervention implementation.

In order to maximize developmental potential and health throughout life, it is important to intervene early in life. Appropriate complementary and/or supplementary feeding for less privileged young children can ensure and improve growth and micronutrient status by providing additional energy and required nutrients. Proper nutrition may also improve immunity and reduce the risk of infection (Barker 1992; Pretince 2005). Through optimal IYCF practices and an integration of knowledge in proper feeding practices, growth complications, infant mortality, and moderate and severe acute child malnutrition can be prevented and child nutrition can be sustained (Dewey 2008) Figure 1.

**Figure 1:**
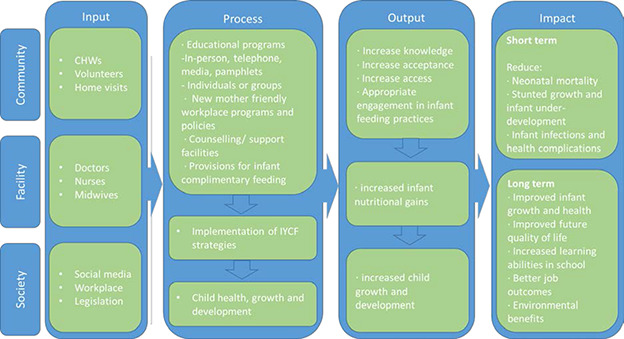
Conceptual framework

Introduction of complementary and supplementary feeding may have some adverse effects as well. The introduction of solids as complementary and supplementary foods means that infants are exposed to microbial contaminated foods (Tang 2015). A lower level of breast milk consumption and/or contaminated foods causing infections may result in infants facing enteric infections, gastrointestinal symptoms (nausea, vomiting, diarrhoea) and decreased energy intake altogether (Romulus‐Nieuwelink 2011). A systematic review in 2013 found evidence suggesting that the very early introduction of solid foods may predispose children to becoming overweight in childhood (Pearce 2013).

### Why it is important to do the review

According to the 2013 Lancet series on maternal and child nutrition, implementation of optimal IYCF practices can prevent an estimated 1.4 million deaths annually among children under five years of age (Black 2013; UNICEF 2011). Moreover, the 2003 Lancet Survival series published rankings of top preventative child survival interventions, with exclusive breastfeeding ranked number one and complementary feeding ranked number three (UNICEF 2011). Various reviews and primary research have been published on IYCF; these show how the interventions have implemented a lasting impact by integrating breastfeeding or complementary feeding within society.

While there are existing reviews examining the effects of various individual interventions promoting optimal breastfeeding and complementary feeding practices, most of those systematic reviews were limited to one or two interventions and focus on either breastfeeding (Abdulwadud 2007; Dyson 2005; Haroon 2013; Lumbiganon 2012; Sinha 2017; Shakya 2017) or complementary feeding (Dewey 2008;Shi 2011; Graziose 2018; Hodder 2017; Lassi 2013). There is also a need for a rigorous review of implementation experiences using facility‐ and community‐based programme interventions to generate evidence for effective delivery approaches for the prevention of acute malnutrition. As existing reviews only focus on a specific population or intervention and included a certain type of study, therefore a broader review of available evidence is strongly needed. The social and environmental constraints imposed by the trial designs in large complex health programs make it difficult at times to use the flexible approaches (such as peer support by male health workers and culturally non acceptable food option) considered fundamental to derive strong conclusions. It is very difficult to meet the requirements of a ‘model’ RCT especially in large‐scale health promotion programs and in public health interventions where interventions may not be under control of an investigator (Audrey 2006). Furthermore, most of those reviews are outdated and require an update (most of the reviews last search results from 2012 and before).

The existing reviews only focused on a specific population or intervention and included a certain type of study (mostly RCTs), therefore a broader review of available evidence is strongly needed. The proposed review will analyse the effectiveness of interventions on outcomes based on different timings and duration of interventions, setting where interventions were employed, and moderators who provided the intervention, which have not been assessed previously. Some reviews only looked at the impact of education intervention for improving the different types of feedings. Therefore, it is important to perform a comprehensive review on all the past research studying interventions to promote IYCF practices and prevent development of SAM/MAM among children living in LMICs. This review will enable an updated and comprehensive assessment of the effectiveness of these interventions that can promote practices for improving child health and nutrition outcomes. The evidence from this systematic review will be critical to inform policy and programmatic decision‐making in initiating directed programs to increase optimal IYCF practices and decrease the prevalence of MAM/SAM in LMICs. Therefore, we aim to systematically review and assess current evidence on the effectiveness of interventions as well as programmes that have been adapted to manage children with acute malnutrition.

## Objectives

The overall objective is to assess the effects of the intervention that aim to promote IYCF practices and their effect on health and nutritional status of children in LMICs. The objectives are to assess the effectiveness of the following on child health and nutritional status:

1. Interventions to promote early and exclusive breastfeeding
2. Interventions to promote continued breastfeeding
3. Interventions to promote appropriate complementary feeding (education or provision of complementary food) during infancy and childhood
4. Effectiveness of community‐based interventions to prevent moderate and severe acute malnutrition

## Methodology

Note: when completing this section, please refer to the Campbell Collaboration Systematic Reviews: Policies and Guidelines. At a minimum, this section should include the information under each of the sub‐sections below:

### Criteria for including and excluding studies

#### Types of study designs

We will include primary studies, including large‐scale programme evaluations to assess the efficacy and/or effectiveness of interventions using the following study designs:

► Randomized controlled trials (RCTs), where participants were randomly assigned, individually or in clusters, to intervention and comparison groups. Cross‐over designs will be eligible for inclusion.
► Quasi‐experimental designs, which include:
  ▷ Natural experiments: studies where non‐random assignment is determined by factors that are out of the control of the investigator. One common type includes allocation based on exogenous geographical variation.
  ▷ Controlled before‐after studies (CBA), in which measures were taken of an experimental group and a comparable control group both before and after the intervention. We also require that appropriate methods were used to control for confounding, such as statistical matching (e.g. propensity score matching, or covariate matching) or regression adjustment (e.g. difference‐in‐differences, instrumental variables).
  ▷ Regression discontinuity designs; here, allocation to intervention/control is based upon a cut‐off score.
  ▷ Interrupted time series (ITS) studies, in which outcomes were measured in the intervention group at least three time points before the intervention and after the intervention.

Pre‐post studies *without* a control group will not be included.

#### Types of participants

The target population for the intervention is mothers/caretakers of children under two2 years of age, regardless of health status, living in LMICs. Both women of reproductive age and pregnant women will be included. However, these interventions are aimed to improve child nutrition and health, so we are interested in child outcomes. We will also include studies that only include a subset of eligible participants and have reported outcomes for the eligible participants separately. Where the study includes a subset of ineligible participants we will exclude those studies and report in reasons for exclusion table.

#### Types of interventions

The following interventions will be included:

► Interventions to promote early initiation and exclusive breastfeeding (such as education and support to mothers to promote early and exclusive breastfeeding)
► Interventions to promote continued breastfeeding (such as education and counselling to mothers)
► Interventions to promote dietary diversification and appropriate complementary feeding (such as education and the provision of complementary food for healthy individuals)
► Interventions for growth monitoring and promotion (training and education to health workers and caregivers). Most growth monitoring programs use weight charts to document child's weight‐for‐age. The promotion interventions can include teaching mothers and guardians, making a contact between mothers and primary health care providers and mobilizing community (Ashworth 2008).
► Interventions to prevent moderate and severe acute malnutrition (education and provision of supplementary food for children suffering from MAM or SAM)

Interventions implemented by healthcare workers, community health workers and through mobile technology platforms will be assessed. Interventions will be compared against no intervention or standard of care (whatever is applicable in the setting the study was conducted.

#### Types of outcome measures

**Primary outcomes**

► Rates of early initiation of breastfeeding (within an hour of birth)
► Rates of exclusive breastfeeding (at 3 and 6 months of age)
► rates of continued breastfeeding (at 12 and 24 months)
► Infant growth (weight gain, height gain, Z‐scores for height‐for‐age, weight‐for‐height, weight‐for‐age, stunting, wasting and underweight)

Both clinical observations and self‐report will be used as methods to assess the rates of breast feeding.

**Secondary outcomes**

► Neonatal mortality (death from any cause within a month of birth among total live births)
► Infant mortality (death from any cause within 12 months of age among total live births)
► Neonatal sepsis (the proportion of neonates dying due to possible serious bacterial infections among all neonates)
► Incidence of acute respiratory infections
► Incidence of diarrhoea
► Any adverse events including gastrointestinal symptoms, noncompliance or decreased feeding
► Cost‐effectiveness of the intervention (it will be reported in narrative text)
► Psychosocial health of the infant (Different scales for psychomotor development, cognitive development, attention, memory, language)

Time points will be plotted within same forest plot with different subgroups for growth and psychosocial outcomes.

Studies will not be excluded if they have not reported the outcomes mentioned above.

#### Types of settings

Studies from LMICS. Country income will be classified according to the 2018 World Bank List of Country Economies (World Bank 2018). Countries which were in the list of LMIC before 2018 (from 2010) would be treated as LMICs.

### Search strategy

We will not impose any restrictions, for example language, date publication status, on the literature searches described below. We will also search for any relevant retraction statements and errata for information.

**Electronic searches**

The search will be performed in the following electronic databases: Cochrane Controlled Trials Register (CENTRAL), MEDLINE, Embase, CINAHL, PsycINFO, ERIC, Sociofiles, HMIS (Health Management Information Consortium), CAB Global Health (https://www.cabi.org/publishing‐products/online‐information‐resources/global‐health/), the WHO nutrition databases (http://www.who.int/nutrition/databases/en/), Popline (https://www.popline.org), Epistemonikos (https://www.epistemonikos.org/en/), Social Science Citation Index, Dissertation Abstracts International, and WHO Global Health Index which covers the WHO Regional journals from Latin America (LILACS), Africa (AFRO) etc. We will also search the websites of selected development agencies or research firms (for example, JOLIS, IDEAS, IFPRI, NBER, USAID, World Bank and Eldis.org). The trials registry Clinicaltrials.gov and WHO's ICRTP will be searched for ongoing trials. Appendix 1

We will use EPOC filters for quantitative design EPOC 2017b.

**Searching other resources**

We will make every effort to contact relevant organizations and experts in the field to identify unpublished or ongoing studies. We will also search for citations at Google Scholar and Web of Sciences. References of included articles, relevant reviews, and annotated bibliographies will be scanned for eligible studies.

### Statistical procedures and conventions

We will conduct data collection and analysis in accordance with the Cochrane Handbook for Systematic Reviews of Interventions (Higgins 2011).

### Selection of studies

Two review authors will independently screen titles and abstracts of all retrieved references. We will retrieve the full‐text study reports for all citations that at least one review author considers potentially relevant. Two review authors will independently screen the full text articles and identify studies for inclusion, and identify and record reasons for exclusion of ineligible studies in a ‘Characteristics of excluded studies’ table. We will include studies irrespective of whether measured outcome data are reported in a ‘usable’ way. We will resolve any disagreement through discussion or, if required, we will consult a third review author. We will identify and exclude duplicates and collate multiple reports of the same study so that each study, rather than each report, is the unit of interest in the review. We will record the selection process in sufficient detail to complete a Preferred Reporting Items for Systematic Reviews and Meta‐Analyses (PRISMA) flow diagram (Moher 2009)

### Data extraction and management

Two review authors will independently extract data on data extraction sheet. In case of any discrepancies in data entry, we will resolve disagreements by consensus or by involving a third review author. We will use a piloted data collection form for study characteristics and outcome data. If any information from the study is unclear or missing or cannot be obtained from the published papers, we will contact the authors of the original papers for further details. We will not impute missing information and will mention those studies as pending under awaiting classification.

Data will be recorded on a data extraction form that will summarise key characteristics of the review/studies including:

► methods: study design, study duration; location/setting of study, sample size, dropout/attrition rate
► details of study participants including their age, location, sample size, numbers randomised, inclusion and exclusion criteria
► interventions: duration of intervention, comparisons, frequency, exposure of intervention, delivery channels for intervention
► outcomes and time point
► information on sample size, methods of analysis, adjustment of confounders, socioeconomic status, gender, age, race/ethnicity, culture, geo‐political region should be collected specifically for quasi experimental designs

If a study is included with more than two intervention arms, we will only include the arms that meet the eligibility criteria.

### Assessment of risk of bias in included studies

Two review authors will independently assess the risk of bias for each included study. We will resolve any disagreements by discussion or by involving a third review author.

For RCTs, including cluster RCTs, we will use the Cochrane Collaboration Risk of Bias tool (Higgins 2011). We will assess the risk of bias according to the following domains. We will justify the categorical risk of bias/study quality judgments (e.g. high, low, and unclear) with information directly from the study.

► Random sequence generation.
► Allocation concealment.
► Blinding of participants and personnel.
► Blinding of outcome assessment for each outcome.
► Incomplete outcome data.
► Selective outcome reporting.
► Other bias such as the validity of outcome measure and baseline comparability.

For non‐randomised controlled trials controlled before‐after studies, and interrupted time series, we will use EPOC methods (EPOC 2017a) to assess the risk of bias according to the following domains. We will justify the categorical risk of bias/study quality judgments (e.g. high, low, and unclear) with information directly from the study.

► Random sequence generation
► Allocation concealment
► Baseline outcome measurements
► Baseline characteristics Incomplete outcome
► Knowledge of the allocated interventions adequately prevented during the study
► Protection against contamination
► Selective outcome reporting
► Other risks of bias

### Measures of treatment effect

We will upload the outcome data for each study into the data tables in RevMan to calculate the treatment effects (RevMan 2014). We will use the risk ratio (RR) for dichotomous outcomes. We will use the mean difference (MD) for continuous outcomes reported on the same scale, and the standardized mean difference (SMD) for continuous outcomes reporting the same outcome but measured on different scales in different studies included in the same meta‐analysis. We will express the uncertainty with 95% confidence intervals (CIs) for all effect estimates. When means and standard deviations are not reported, we will use other available data (for example, confidence intervals, t values, P values) and appropriate methods described in the Cochrane Handbook for Systematic Reviews of Interventions (Higgins(b) 2011) to calculate the means and standard deviations. Where other available data are not sufficient to calculate standard deviations, we will contact the study authors. When we are unable to enter the results in either way, we will describe them in the ‘Characteristics of included studies' tables or enter the data into the ‘Additional tables’ section. We will also consider the possibility and implications of skewed data when analysing continuous outcomes as they can mislead results due to small sample size. We will analyze outcomes from studies with multiple groups in an appropriate way to avoid double counting of participants by adding them to different subgroups within same plot. In such scenario, we will not report the overall pooled estimate and we will only report subgroup pooled estimate.

### Unit of analysis issues

We have a number of different outcomes and outcome subcategories. Conceptually, these subcategories cannot be combined (for example, within the cognitive development, language cannot be combined with intelligence). Therefore, a meta‐analysis will be conducted separately for each outcome. Furthermore, for each outcome, we will separately meta‐analyse different study designs (ITS, RCT, and CBA). We will report all the effect sizes for each outcome and will not prioritise any from others.

Where trials have used clustered randomizations, we anticipate that study investigators would have presented their results after appropriately controlling for clustering effects (for example, variance inflated standard errors, hierarchical linear models). If it is unclear whether a cluster‐ randomised controlled trial has appropriately accounted for clustering, the study investigators will be contacted for further information. Where appropriate controls for clustering were not used, we will request an estimate of the intra‐class correlation coefficient (ICC). Following this, effect sizes and standard errors will be meta‐analysed in RevMan using the generic inverse method (Higgins(b) 2011). They will be combined with estimates from individual‐level trials.

### Dealing with missing data

We will contact trial authors to verify key study characteristics and obtain missing numerical outcome data where possible (e.g. when we identify a study as an abstract only). If we do not find a full report even after we contact the study authors, we will list such an abstract as a ‘study awaiting classification’. If numerical outcome data are missing, such as standard deviations (SDs) or correlation coefficients, and we cannot obtain these from the study authors, we will calculate them from other available statistics, such as P values, according to the methods described in the Cochrane Handbook for Systematic Reviews of Interventions (Higgins 2011).

### Assessment of heterogeneity

Statistical heterogeneity will be assessed using Tau^2^, I^2^ and significance of the Chi‐square test; we will also assess heterogeneity visually using forest plots. Based on prior theory and clinical knowledge, we expect clinical and methodological heterogeneity in effect sizes in this literature. Therefore, we will attempt to explain any observed statistical heterogeneity using subgroup analysis.

### Assessment of reporting biases

If sufficient studies are found, funnel plots will be drawn to investigate any relationship between effect size and study precision. Ten studies are usually considered sufficient to draw a funnel plot. As a direct test for publication bias, we will compare results extracted from published journal reports with results obtained from other sources (including correspondence). Whilst funnel plot asymmetry may indicate publication bias, this is not inevitably the case, and possible explanations for any asymmetry found will be considered and discussed in the text of the review.

### Data synthesis

We will carry out statistical analysis using RevMan 2014 software.

We will prepare a matrix of all studies for each intervention which will outline all the differences in the studies at the intervention, duration, timing etc. and examine how to pool them together. We expect that most of our meta‐analyses will be random‐effects meta‐analyses, given the diverse contexts, participants, interventions etc.

For each comparison, we will descriptively summarise the findings from the contextual factors such as setting, timings of intervention, duration of intervention, people delivering interventions etc. to assess their impact on the implementation and effectiveness of each intervention.

### ‘Summary of findings’ tables

We will construct ‘Summary of findings’ tables for all of the primary outcomes using the Grading of Recommendations Assessment, Development and Evaluation (GRADE) criteria (Guyatt 2011) which involves consideration of within‐study risk of bias (methodological quality), directness of evidence, heterogeneity, precision of effect estimates and risk of publication bias. We will rate the quality of the body of evidence for each key outcome as ‘high’, ‘moderate’, ‘low’ or ‘very low’. Non‐randomized studies will initially be rated as ‘low’ quality. If there are no serious methodological flaws, we will upgrade the evidence for studies with a large magnitude of effect; presence of a dose response relationships; and effect of plausible residual confounding.

We will GRADE and prepare the following summary of findings tables

Breastfeeding

► Rates of early initiation of breastfeeding (within an hour of birth)
► Rates of exclusive breastfeeding (at 3 and 6 months of age)
► rates of continued breastfeeding (at 12, 18 and 24 months)

Complementary feeding

► Mean Z score for height‐for‐age
► Mean Z score for weight‐for‐height
► Mean Z score for weight‐for‐age
► Stunting
► Wasting
► Underweight

Supplementary feeding

► Prevalence of malnutrition
► Mean Z score for height‐for‐age
► Mean Z score for weight‐for‐height
► Mean Z score for weight‐for‐age
► Stunting
► Wasting
► Underweight

### Subgroup analysis and investigation of heterogeneity

We will conduct the following subgroup analyses when there is a sufficient number of studies in each subgroup.

► Setting (home, facility‐based, community level): to see the effectiveness of interventions on the outcomes based on the settings where those were provided
► Timing of intervention (prenatal, natal, post‐partum): to assess the effectiveness of interventions when it was delivered
► Duration of intervention: to assess the effectiveness of different durations of intervention
► Self‐reported or clinically observed
► Recall information from last 24 hours or from birth.
► Moderators (People) delivering the interventions and contextual factors
  ▷ Type of health workers (Community health workers (CHW), physicians, nurses, midwives, volunteers, counsellors): to assess the most effective mode of intervention distribution
  ▷ Training and supervision of health workers: to assess the most effective mode of intervention distribution.

The subgroup analyses will be conducted using Review Manager 5.3 with a test for interaction.

### Sensitivity analysis

Sensitivity analyses will be performed to consider the impact of the following.

► Allocation concealment (adequate versus inadequate and/or unclear).
► Attrition (< 10% versus ≥ 10%).

Imputed inter correlation coefficients that have been derived in different ways.

### Treatment of qualitative research

We do not plan to include qualitative research.

## Review authors

**Lead review author:** The lead author is the person who develops and co‐ordinates the review team, discusses and assigns roles for individual members of the review team, liaises with the editorial base and takes responsibility for the on‐going updates of the review.

**Table:** 

Name: Zohra Lassi	Zohra Lassi
Title:	NHMRC Public Health and Health Services Fellow
Affiliation:	Robinson Research Institute, University of Adelaide
Address:	Room 132, Level 1, Helen Mayo North, 30 Frome Road
City, State, Province or County:	Adelaide, South Australia, Australia
Post code:	5005
Country:	Austalia
Phone:	+61883139266
Email:	Zohra.lassi@adelaide.edu.au
**Co‐authors:**	
Name:	Omar Irfan
Title:	Research Associate
Affiliation: Aga Khan University	Affiliation: Aga Khan University
Address: Stadium Road	Address: Stadium Road
City, State, Province or County: Karachi, Sindh	City, State, Province or County: Karachi, Sindh
Post code: 74800	Post code: 74800
Country: Pakistan	Country: Pakistan
Phone: +92.21.3486.4717	Phone: +92214867474
Email: jai.das@aku.edu	Email: omarirfan1@hotmail.com
	
Name: Rabia Hadi	
Title: Research Officer	
Affiliation: Aga Khan University	
Address: Stadium Road	
City, State, Province or County: Karachi, Sindh	
Post code: 74800	
Country: Pakistan	
Phone: +92.21.3486.4717	
Email: hadirabia@gmail.com	
	
Name: Jai Das	
Title: Assistant Professor	
Affiliation: Aga Khan University	
Address: Stadium Road	
City, State, Province or County: Karachi, Sindh	
Post code: 74800	
Country: Pakistan	
Phone: +92.21.3486.4717	
Email: jai.das@aku.edu	
Name: Zulfiqar A. Bhutta	
Title: Co‐Director, Centre for Global Child Health	
Affiliation: The Hospital for Sick Children	
Address: 686 Bay Street, 11^th^ Floor	
City, State, Province or County: Toronto, Ontario	
Post code: M5G 0A4	
Country: Canada	
Phone: +1.416.813.7654	
Email: zulfiqar.bhutta@sickkids.ca	

## Roles and responsibilities

- Content: Zohra Lassi, Jai Das, Zulfiqar Bhutta
- Systematic review methods: Omar Irfan, Zohra Lassi, Jai Das
- Statistical analysis: Omar Irfan, Zohra Lassi, Jai Das
- Information retrieval: Rabia Hadi, Omar Irfan, Zohra Lassi

## Sources of support

Funding for this review came from a grant from the Bill & Melinda Gates Foundation to the Centre for Global Child Health at The Hospital for Sick Children (Grant No. OPP1137750)

## Declarations of interest

Rabia Hadi and Omar Irfan: None to declare.

Zohra Lassi, Jai Das and Zulfiqar Bhutta have published the following reviews:

Lassi ZS, Das JK, Zahid G, Imdad A, Bhutta ZA. Impact of education and provision of complementary feeding on growth and morbidity in children less than 2 years of age in developing countries: a systematic review. BMC Public Health. 2013;13 Suppl 3:S13.

Imdad A, Yakoob MY, Bhutta ZA. Impact of maternal education about complementary feeding and provision of complementary foods on child growth in developing countries. BMC Public Health2011;11 (Suppl 3) :S25

Imdad A, Yakoob MY, Bhutta ZA. Effect of breastfeeding promotion interventions on breastfeeding rates, with special focus on developing countries. BMC Public Health2011; 11 (Suppl 3) :S24

Haroon S, Das JK, Salam RA, Imdad A, Bhutta ZA. Breastfeeding promotion interventions and breastfeeding practices: a systematic review. BMC Public Health201313 (Suppl 3) :S20

## Preliminary timeframe

Approximate date for submission of the systematic review: July 2019

Please note this should be no longer than two years after protocol approval. If the review is not submitted by then, the review area may be opened up for other authors.

## Plans for updating the review

Reviews should include in the protocol specifications for how the review, once completed, will be updated. This should include, at a minimum, information on who will be responsible and the frequency with which updates can be expected.

Zohra S Lassi will be responsible for updating this review and the review will be update every two years after publication date.

## AUTHOR DECLARATION

### Authors' responsibilities

By completing this form, you accept responsibility for preparing, maintaining and updating the review in accordance with Campbell Collaboration policy. Campbell will provide as much support as possible to assist with the preparation of the review.

A draft review must be submitted to the relevant Coordinating Group within two years of protocol publication. If drafts are not submitted before the agreed deadlines, or if we are unable to contact you for an extended period, the relevant Coordinating Group has the right to de‐register the title or transfer the title to alternative authors. The Coordinating Group also has the right to de‐register or transfer the title if it does not meet the standards of the Coordinating Group and/or Campbell.

You accept responsibility for maintaining the review in light of new evidence, comments and criticisms, and other developments, and updating the review at least once every five years, or, if requested, transferring responsibility for maintaining the review to others as agreed with the Coordinating Group.

### Publication in the Campbell Library

The support of the Coordinating Group in preparing your review is conditional upon your agreement to publish the protocol, finished review, and subsequent updates in the Campbell Library. Campbell places no restrictions on publication of the findings of a Campbell systematic review in a more abbreviated form as a journal article either before or after the publication of the monograph version in Campbell Systematic Reviews. Some journals, however, have restrictions that preclude publication of findings that have been, or will be, reported elsewhere and authors considering publication in such a journal should be aware of possible conflict with publication of the monograph version in Campbell Systematic Reviews. Publication in a journal after publication or in press status in Campbell Systematic Reviews should acknowledge the Campbell version and include a citation to it. Note that systematic reviews published in Campbell Systematic Reviews and co‐registered with Cochrane may have additional requirements or restrictions for co‐publication. Review authors accept responsibility for meeting any co‐publication requirements.

**I understand the commitment required to undertake a Campbell review, and agree to publish in the Campbell Library. Signed on behalf of the authors**:

**Table:** 

**Form completed by: Zohra Lassi**	**Date: Dec 6, 2018**

## Appendix

Appendix A

Search strategy

We will prepare a search strategy using the following terms including: breastfeeding, complementary feeding, infant and young child feeding, growth monitoring etc.

**EMBASE** (Embase has specific index terms (Emtree) which are not always the same as MeSH terms, and need to be used)

1) “breast feeding”/de OR “infant feeding”/de OR “breast feed*”:ti,ab OR breastfeed*:ti,ab OR breastfed:ti,ab OR “breast fed”:ti,ab OR “complementary feed*”:ti,ab OR “supplementary feed*”:ti,ab OR “complementary (is this on its own ‐ complementary feed* is therefore unnecessary?)
2) counselling:ti,ab OR intervention*:ti,ab OR “intervention strateg*”:ti,ab OR “growth monitor*”:ti,ab OR “mother* education”:ti,ab OR education:ti,ab OR “prenatal care”/de OR “prenatal care”:ti,ab OR “antenatal care”:ti,ab OR (any more terms here?)
3) child*:ti,ab OR infan*:ti,ab OR newborn*:ti,ab OR neonate*:ti,ab OR baby:ti,ab OR babies:ti,ab OR kid:ti,ab OR kid*:ti,ab OR toddler*:ti,ab OR “preschool child”/de OR infant/exp OR toddler/de OR (more terms?)
4) 1 and 2 and 3
5) limit 4 to human

**PUBMED** (You state in the search section you will search Medline rather than Pubmed ‐ there are differences, so which is it to be?)

1) “breast feeding”[mh:noexp] OR “breast feed*”[tiab] OR breastfeed*[tiab] OR “breast fed”[tiab] OR breastfed[tiab] OR “complementary feed*”[tiab] OR “supplementary feed*”[tiab] OR “complementary food*”[tiab] OR “supplementary food*”[tiab] OR weaning[mh] OR wean*[tiab] OR “infant feed*”[tiab] OR “young child feed*”[tiab] OR “infant food*”[tiab] OR “young child food*”[tiab](Probably a good idea to use proximity/adjacency operators here if you are using Medline ‐ to retrieve infant feeding, feeding of infants etc)
2) Counselling[mh:noexp](Counseling in MeSH ‐ one L) OR counselling[tiab] OR intervention*[tiab] OR “intervention strateg*”[tiab] (not necessary as you have intervention on its own) OR “growth monitor*”[tiab] OR “mothers/education”[mh] OR “mother's education”[tiab] OR education[tiab] OR “prenatal care”[mh] OR “prenatal care”[tiab] OR “antenatal care”[tiab] OR ante natal care”[tiab] OR “pre natal care”[tiab] OR counseling[tiab]
3) Infant[mh] (there are more specific MeSH terms for Infant such as Infant, Newborn, Infant, Low Birth Weight, Infant, Premature etc)OR “child, preschool”[mh] OR child*[tiab] OR infan*[tiab] OR newborn*[tiab] OR neonate*[tiab] OR baby[tiab] OR babies[tiab] OR kid[tiab] OR kid*[tiab] OR toddler*[tiab]
4) 1 and 2 and 3
5) limit 4 to humans (For both these searches there is no study designs filter which you state will be derived from the EPOC filters ‐ this needs to be included here. Also ‐ you state that the review will be concerning infants and under 2s in LMICs, but there is no LMICs filter included in these search strategies) These strategies are unfinished and more work needs to be done.
